# Recombination suppression and evolutionary strata around mating‐type loci in fungi: documenting patterns and understanding evolutionary and mechanistic causes

**DOI:** 10.1111/nph.17039

**Published:** 2020-12-01

**Authors:** Fanny E. Hartmann, Marine Duhamel, Fantin Carpentier, Michael E. Hood, Marie Foulongne‐Oriol, Philippe Silar, Fabienne Malagnac, Pierre Grognet, Tatiana Giraud

**Affiliations:** ^1^ Ecologie Systematique Evolution Batiment 360 Université Paris‐Saclay CNRS AgroParisTech Orsay 91400 France; ^2^ Ruhr‐Universität Bochum, Evolution of Plants and Fungi ‐ Gebäude ND 03/174 Universitätsstraße 150, 44801 Bochum Germany; ^3^ Biology Department, Science Centre Amherst College Amherst MA 01002 USA; ^4^ Mycologie et Sécurité des Aliments MycSA INRAE Villenave d’Ornon 33882 France; ^5^ Lab Interdisciplinaire Energies Demain Univ Paris Diderot Sorbonne Paris Cite Paris 13 F‐75205 France; ^6^ Institute for Integrative Biology of the Cell (I2BC) Université Paris‐Saclay CEA CNRS Gif‐sur‐Yvette 91198 France

**Keywords:** *Agaricus bisporus*, ascomycetes, basidiomycetes, *Cryptococcus*, *Microbotryum*, *Neurospora tetrasperma*, *Podospora anserina*, sex chromosomes

## Abstract

Genomic regions determining sexual compatibility often display recombination suppression, as occurs in sex chromosomes, plant self‐incompatibility loci and fungal mating‐type loci. Regions lacking recombination can extend beyond the genes determining sexes or mating types, by several successive steps of recombination suppression. Here we review the evidence for recombination suppression around mating‐type loci in fungi, sometimes encompassing vast regions of the mating‐type chromosomes. The suppression of recombination at mating‐type loci in fungi has long been recognized and maintains the multiallelic combinations required for correct compatibility determination. We review more recent evidence for expansions of recombination suppression beyond mating‐type genes in fungi (‘evolutionary strata’), which have been little studied and may be more pervasive than commonly thought. We discuss testable hypotheses for the ultimate (evolutionary) and proximate (mechanistic) causes for such expansions of recombination suppression, including (1) antagonistic selection, (2) association of additional functions to mating‐type, such as uniparental mitochondria inheritance, (3) accumulation in the margin of nonrecombining regions of various factors, including deleterious mutations or transposable elements resulting from relaxed selection, or neutral rearrangements resulting from genetic drift. The study of recombination suppression in fungi could thus contribute to our understanding of recombination suppression expansion across a broader range of organisms.

**Table:** 

	Contents	
	Summary	2470
I.	Introduction: Recombination suppression around genes controlling mating compatibility in various organisms	2471
II.	Gene linkage at mating‐type loci in fungi ensures proper nonself‐recognition function	2473
III.	Incorporation at mating‐type loci of genes without function in mating compatibility but involved in mating, without clear benefit of linkage	2476
IV.	Uniparental mitochondrion inheritance or selfing mating systems can trigger beneficial recombination suppression around fungal mating‐type loci	2476
V.	Further recombination suppression beyond fungal mating‐type loci without obvious benefits	2477
VI.	Consequences of recombination suppression: genomic degeneration	2482
VII.	Ultimate and proximate mechanisms generating evolutionary strata beyond sexual antagonism or selection for mating‐type compatibility or mitochondrion inheritance	2482
VIII.	Conclusion and future directions	2487
	Acknowledgements	2487
	References	2487

## I. Introduction: Recombination suppression around genes controlling mating compatibility in various organisms

As a fundamental feature of sexual reproduction, recombination between different genotypes can generate beneficial allelic combinations and purge deleterious mutations (Otto, 2009). The genomic regions involved in promoting such sexual genetic exchange often have evolved extensive recombination suppression. Examples of such genomic regions include sex chromosomes*, self‐incompatibility* loci in plants and mating‐type* loci in fungi and algae (see Box 1 for definitions; Uyenoyama, 2005; Beukeboom & Perrin, 2014; Idnurm *et al*., 2015; Charlesworth, 2016). The most extensively studied among these are sex chromosomes, defined as pairs of chromosomes governing sex determination in many organisms that have separate sexes*. In heterothallic fungi, mating‐type loci are incompatibility loci not associated with gamete size determinism (i.e. not associated with separate sexes*); successful mating is possible only between gametes or cells carrying different alleles at the mating‐type loci based on molecular nonself‐recognition, regardless of gamete or cell size. The suppression of recombination at the loci determining sexes or mating types maintains the allelic combinations at two or more genes required for correct sex or mating‐type functions (e.g. genes responsible for induction of male* function and inhibition of female* function or mating‐type pheromone and pheromone receptor genes; Charlesworth, 2017). This represents a case of beneficial allelic associations maintained at multiple genes through recombination suppression, which are called ‘supergenes’ (Charlesworth, 2016).

Box 1 **Glossary (terms defined below are indicated by an asterisk in the text)**

**Anisogamy:** trait of a population or a species in which mating only occurs between gametes of different sizes.

**Antagonistic selection:** situation where different morphs, sexes or phases have different fitness optima; a particular case of antagonistic selection is sexual antagonism (i.e. different fitness optima between sexes for traits other than sex determinism or gamete compatibility), and another case could be mating‐type antagonism (i.e. different fitness optima between mating types for traits other than mating‐type determinism).

**Automixis:** particular form of selfing, in which mating or nuclear combinations occur among products of a single meiosis.

**Dikaryotic:** in most basidiomycetes, there is an extended dikaryotic phase –with two separate haploid nuclei per cell, karyogamy taking place only just before meiosis. In ascomycetes, this dikaryotic stage is very brief and quickly followed by karyogamy. In some cases, there may be more than two nuclei per cell.

**Female gamete:** large haploid cell that will mate with a small, often mobile or dispersing, haploid cell (male gamete) to form a zygote.

**Heterothallism:** trait of a population or a species in which mating can only occur between different mating types, as opposed to homothallism*. This definition is based on experimental observations *in vitro*, when a single haploid strain is unable to mate with itself; this does not, however, inform on the mating system in natural populations (diploid selfing vs outcrossing). *Microbotryum* fungi, for example, are heterothallic and highly selfing.

**Hermaphrodite:** Individual able to produce both male* and female* gametes.

**Hemizygous:** character of a gene present in one of the sex or mating‐type chromosomes* but absent from the other sex or mating‐type chromosome, often maintained under sheltered permanent heterozygosity.

**Heterogametic:** situation of a sex chromosome that is always found in a heterozygous state (e.g. the Y chromosome in XY males).

**Homogametic:** situation of a sex chromosome that can be found in a homozygous state (e.g. the X chromosome in XX females).

**Homothallism:** trait of a population or a species in which mating is not restricted by mating‐type genes, as opposed to heterothallism*. This definition is based on experimental observations *in vitro*, when a single haploid strain is able to mate with itself; this breeding system does not however inform on the mating system in natural populations (diploid selfing versus outcrossing).

**Hyphae:** the filamentous body structure of most fungi.

**Isogamy:** trait of a population or a species in which compatible gametes have the same size.

**Male gamete:** small, often mobile or dispersing haploid, unicellular, cell that will mate with a large haploid cell (female* gamete) to form a zygote.

**Mating‐type chromosomes:** pair of chromosomes determining mating types but not sexes; mating‐type chromosomes are important to distinguish from sex chromosomes as different evolutionary forces may act on these two types of chromosomes determining sexual compatibility. In particular, mating‐type chromosomes determine mating types and not sexes (i.e. male* vs female*) and often lack a homogametic* condition in the diploid stage – they are always heterogametic*.

**Mating‐type locus:** a locus in a genome controlling mating compatibility based on molecular haploid nonself‐recognition, mating being successful only between different alleles, without relationship with gamete size (i.e. without association with male* vs female* functions).

**Pseudo‐homothallic:** trait of a population or a species in which a meiotic reproductive cycle can occur in a culture of a single, pure strain *in vitro*, similar to a homothallic* fungus; but, unlike a homothallic* fungus, the reason for their self‐compatibility is that the spore produced after meiosis is dikaryotic* and heterozygous at the mating‐type locus*. In general, such fungi mate by automixis* *–* fusion among products of a single meiosis, although also they may sometimes outcross in nature.

**Self‐incompatibility locus in plants:** a locus, often encompassing several linked genes, controlling mating compatibility based on molecular haploid or diploid nonself‐recognition, mating being successful only between different alleles; self‐incompatibility loci in plants correspond to a specific case of mating‐type loci and typically occurs in hermaphroditic* plants.

**Separate sexes:** situation in a population or species, also called dioecy or gonochorism, in which individuals are either male* or female* –individuals can only produce either male* or female* gametes, as opposed to hermaphrodites* in which individuals are able to produce both male* and female* gametes.

**Sex chromosomes:** pair of chromosomes determining sex (i.e. male* vs female* individuals).

**Sexual antagonism:** situation where different sexes have different fitness optima, as for example when a trait is selected that enhances transmission through males (e.g. bright color or long tail conferring sexual attractiveness), whereas the trait would be detrimental to transmission through females (e.g. the same trait increasing risk from predation).

**Spore:** in fungi, a spore is the unit of sexual or asexual reproduction consisting of one or a small number of cells that may be adapted for dispersal, survival or mating.

**Syngamy**: union of gametes during the process of sexual reproduction.

**Fig. 1:**
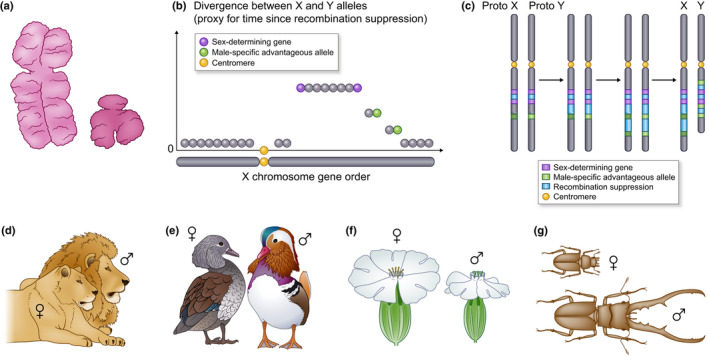
Evolutionary theory based on sexual antagonism to explain evolutionary strata on sex chromosomes. Illustration of sex chromosomes in humans (a), of a typical evolutionary strata pattern on sex chromosomesas found in many plants and animals (b), with blocks of decreasing divergence between the alleles associated with the two sex chromosomes further from the sex‐determining genes when plotted against the ancestral (X) gene order, of sexual dimorphism in lions (d) and birds (e), plants (f) and insects (g), and of the hypothesis of sexual antagonism (c) for explaining evolutionary strata: the green genes with alleles beneficial in only one sex are selected for being linked to the sex‐determining genes in purple, leading to suppressed recombination in blue.

A prominent and consequential feature of some sex chromosomes* is that the regions lacking recombination often extend well beyond the genes directly determining sex, and there can be several successive steps of recombination suppression. In such cases, the initial recombination cessation event at the key sex‐determining genes is followed by the linkage of adjacent chromosome regions at different times, in a stepwise manner, resulting in several adjacent ‘evolutionary strata’ of differentiation between nonrecombining sex chromosomes* (Fig. 1a). Such strata have been identified on many plant and animal sex chromosomes* (Nicolas *et al*., 2005; Bergero & Charlesworth, 2009; Furman *et al*., 2020), by plotting allele divergence between the two sex chromosomes*, used as a proxy for time since recombination suppression (Fig. 1b). Indeed, the alleles at genes linked to the sex‐determining locus accumulate mutations independently with time as soon as they stop recombining. Differentiation between alleles typically is plotted against the ancestral gene order as inferred from the recombining sex chromosome (e.g. the X chromosome in mammals; Fig. 1a,b). The gene order on the nonrecombining sex chromosome (e.g. the Y chromosome) is indeed often highly rearranged, whereas the X–X recombination in females maintains gene order, facilitating tracing of the history of evolutionary steps (Bergero & Charlesworth, 2009).

The reason why recombination suppression extends progressively outward from the sex‐determining genes remains debated (Ironside, 2010; Charlesworth, 2018; Ponnikas *et al*., 2018; Bergero & Charlesworth, 2019; Bergero *et al*., 2019). The prevailing hypothesis is the influence of sexually antagonistic selection*, according to which a suite of genes with alleles beneficial in one sex but deleterious in the other become successively linked to the sex‐determining locus (Fig. 1c) (Bergero & Charlesworth, 2009; Charlesworth, 2017). Alleles beneficial only to males but deleterious in females – for instance, brighter coloration utilized in mate attraction under female* choice – would be selected for linkage to the male‐determining allele (Fig. 1d,e). Sexual antagonism* provides an attractive and theoretically plausible explanation for the existence of evolutionary strata on sex chromosomes*, but decades of research on diverse animals and plants have provided little conclusive empirical evidence to support it (Beukeboom & Perrin, 2014; Wright *et al*., 2016). Alternative hypotheses have been proposed, including some unrelated to any positive selection on functions in association with different sexes, such as the following hypotheses: (1) the successive linkage of genes accumulating deleterious recessive mutations on the heterogametic* sex chromosome due to linkage disequilibrium with sex‐determining loci, where complete linkage ensures heterozygosity and thereby permanent sheltering of genetic load mutations (Antonovics & Abrams, 2004); (2) genetic drift, or positive selection unrelated to sexual antagonism, fixing chromosomal rearrangements suppressing recombination (Ironside, 2010; Ponnikas *et al*., 2018; Olito & Abbott, 2020); and (3) transposable element (TE) accumulation in nonrecombining regions, resulting from relaxed selection, driving the expansion of recombination suppression into adjacent regions through the impacts of TE genomic silencing by DNA methylation or chromatin modifications (heterochromatinization) (Kent *et al*., 2017). These hypotheses have been very little studied but may play an important role in the evolution of sex‐related chromosomes, even if they are not necessarily exclusive of potential effects of sexual antagonism*.

Although mating‐type loci in fungi have been the subject of extensive functional studies, their evolutionary aspects remain little investigated despite their numerous assets as experimental and genetic models, and the notable similarities to canonical sex chromosomes* in animals and plants. It has long been known that mating‐type loci of fungi have incorporated genes not involved in mating‐type determination. However, these phenomena in fungi have not generally been placed within the broader evolutionary theory of recombination suppression on sex chromosomes*, and evolutionary biologists often know little about life history and mating aspects of fungi. Many fungi are heterothallic*, meaning that mating can only occur between different mating types, with cell compatibility being determined in the haploid stage. However, fungi are not likely to be widely affected by sexual antagonism*, as they do not have separate sexes* – thus, they do not generally have individuals that are male* or female*. Actually, many fungi are isogamous*, without size‐differentiated male* and female* gametes. Even in fungi producing small and large gametes or undergoing sex by mating between a spore* and a hypha* as a form of anisogamy*, gamete size is not determined by the mating‐type locus*; all haploid genotypes are hermaphrodites*, being able to produce male* and female* gametes, whereas compatibility is most often still determined by molecular mechanisms controlled by the mating‐type loci (Butler, 2007; Fraser *et al*., 2007; Billiard *et al*., 2011, 2012). Basidiomycete fungi do not produce small and large differentiated gametes; however, in some species such as *Schizophyllum commune*, male‐like and female‐like roles can still be distinguished during mating: the female‐like role is played by the haploid mycelium as being a nucleus‐acceptor and the male‐like role by a spore* falling on the mycelium as the nucleus‐donor (Kronstad & Staben, 1997; Butler, 2007; Fraser *et al*., 2007; Nieuwenhuis *et al*., 2011; Nieuwenhuis & Aanen, 2012). In such cases, too, all haploid genotypes are able to play male‐like and female‐like roles. Anisogamy* in fungi, when it exists, is thus decoupled from mating‐type determinism, so that stepwise extension of recombination suppression around mating‐type genes, when present, cannot result from sexual antagonism*. This decoupling of the cessation of recombination from sexual antagonism* allows the testing of alternative hypotheses, which has only been recognized recently (Branco *et al*., 2017).

The utility of fungi as tractable models stems in part from the mating type in heterothallic* species being determined at the haploid stage, mating being only successful between cells carrying different alleles at the mating‐type locus/loci* (Kronstad & Staben, 1997; Feldbrugge *et al*., 2004; Butler, 2007; Fraser *et al*., 2007). The haploid stage often is culturable, allowing easy access to valuable developmental, genetic and genomic analyses, especially as they often have small and compact genomes. In ascomycetes (molds), mating type is determined by a single locus. In most basidiomycetes (mushrooms, rusts and smuts), mating type is determined by two loci, often located on different chromosomes (Coelho *et al*., 2017). In basidiomycetes, the pheromone‐receptor (PR) locus controls the compatibility at the pre‐mating stage (i.e. for initiating syngamy), whereas the mating‐type homeodomain (HD) locus controls post‐mating sexual compatibility. Mating‐type loci have typically two alleles in fungi, although some groups such as the Agaricomycetes (including many mushrooms) have evolved multiple alleles (Casselton & Kues, 2007; James, 2015).

We review below the evidence for recombination suppression at mating‐type loci focusing mainly on heterothallic* fungi: first at the mating‐type genes themselves, and then in the cases where recombination has extended into adjacent chromosomal regions, or incorporated additional genes from elsewhere in the genome, and we discuss the possible ultimate and proximate causes of such evolutionary strata, as well as their evolutionary consequences in terms of genomic degeneration. There have been many reviews and studies on functional aspects of fungal mating‐type loci, but studies on the patterns and evolutionary causes of recombination suppression extending farther than mating‐type genes remain too scarce. We hope that recent findings highlighted here will foster further research on the expansion of recombination suppression around mating‐type loci in fungi, to assess the generality of these patterns, and to test hypotheses about the evolutionary genomics of reproductive compatibility across a broader range of organisms.

## II. Gene linkage at mating‐type loci in fungi ensures proper nonself‐recognition function

**Fig. 2:**
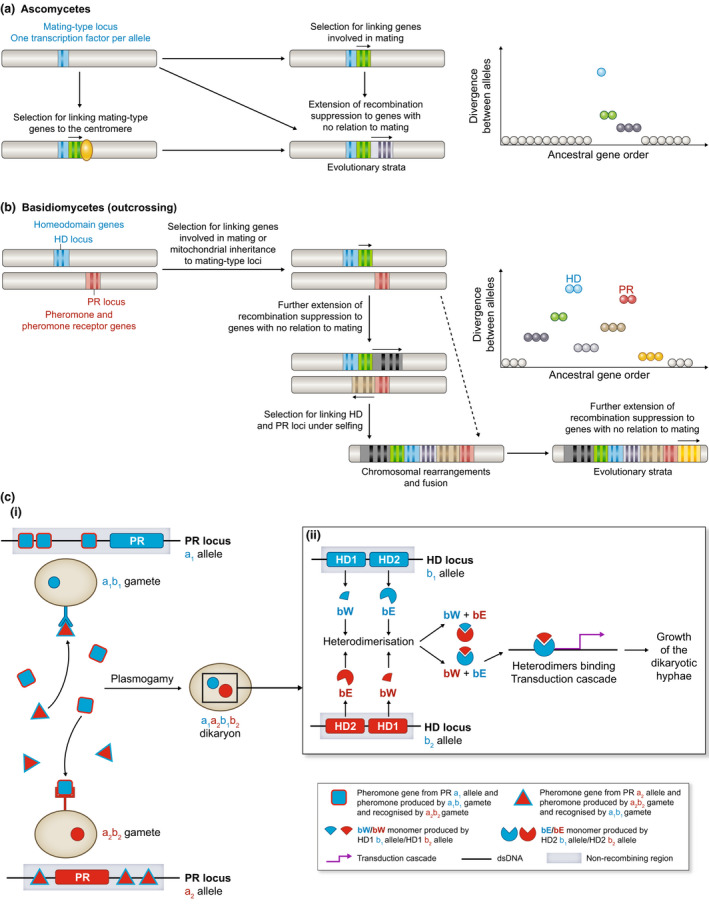
Illustration of recombination suppression at mating‐type loci in fungi and extending beyond, forming evolutionary strata of differentiation between mating‐type chromosomes (see main text for details) in (a) ascomycetes and (b) in basidiomycetes. (c) Functions of mating‐type genes in basidiomycetes. Successful mating is only possible between haploid cells carrying different alleles at each of the PR and HD locus. (i) Mating between a a_1_b_1_ gamete and a a_2_b_2_ gamete. The PR locus is involved in gamete recognition and syngamy. In each mating type, the pheromone receptor allele is linked to its incompatible pheromone allele. (ii) The HD locus is involved in the maintenance and growth of the dikaryotic* hyphae*. This function is achieved by a transcriptional regulation resulting from the dimerization of two homeodomain proteins produced by alternative alleles from distinct HD genes.

Recombination suppression at mating‐type loci has been long recognized in fungi for maintaining function of distinct alleles in the haploid nonself‐recognition system (Kronstad & Staben, 1997; Butler, 2007; Fraser *et al*., 2007; Stankis & Specht, 2007) (Fig. 2a,b; Table 1). Because mating type in heterothallic* species is determined at the haploid stage, as in brown algae or mosses (Ahmed *et al*., 2014), all diploid or dikaryotic* individuals are heterozygous (i.e. there is no homogametic* state as, for example, in the mammalian system with homogametic* XX females and heterogametic* XY males). In ascomycete fungi, the mating‐type locus* encodes transcription factors that control the mating‐type‐specific pheromone and pheromone‐receptor genes located elsewhere in the genome. The mating‐type locus* in ascomycetes also controls the expression of many other genes in the genome, some being involved in the mating process (Bidard *et al*., 2011; Coppin *et al*., 2012). Although in a comparable genomic location, the alternate mating‐type alleles are so different between mating types that they are called idiomorphs rather than alleles and often are considered as nonhomologous. However, it may be that the alleles have differentiated so much as a consequence of a very ancient recombination suppression that their homology is no longer recognizable (Debuchy & Turgeon, 2006). In many homothallic* ascomycetes (i.e. with no discrimination based on mating type for compatible mates), the two mating‐type alleles are present and closely linked in each haploid genome (Lin & Heitman, 2007), which may be the result of some kind of recombination suppression, although this has not been studied; one does not expect the suppression of homologous recombination between parental genomes within mating‐type genes in homothallic fungi as all haploid individuals carry the same alleles, but one may expect selection against heterologous recombination between the two idiomorphs (if/when they were still similar enough for recombination between them to be possible) that would yield progeny with unbalanced number of genes.

**Table 1:** Possible evolutionary (ultimate) and mechanistic (proximate) causes for recombination suppression at mating‐type loci in fungi.

**Ultimate causes**
*Proper nonself‐recognition:* functions directly involved in nonself‐recognition.
*Mating‐type antagonism*: functions that may possibly improve the fitness of one mating type but not the other mating type but that would not be related directly to the nonself‐recognition function *per se;* for example, a gene increasing the production or attractiveness of a single pheromone allele (e.g. the duplication in several copies of pheromone genes).
*Selection for associating a function other than nonself‐recognition to the mating‐type function*: e.g. mitochondrion uniparental inheritance.
*Selection for increasing mating compatibility odds:* for example, linkage between the two mating‐type loci in basidiomycetes, or between mating‐type loci and centromeres, or for inducing a single crossing‐over between the mating‐type locus and the centromere.
*Selection for co‐regulation:* it may be beneficial to use the same promoters as mating‐type determining genes for genes that need to be expressed only during mating. *Selection for sheltering deleterious alleles:* if deleterious alleles accumulate in the margin of the mating‐type locus due to partial sheltering, selection for sheltering them permanently in a heterozygous state by complete recombination cessation may be selected for (Fig. 8a)
*Genetic drift fixing neutral inversions/rearrangements:* rearrangements at the margin of the mating‐type locus may be neutral and fixed by genetic drift, but can only be fixed in the mating‐type chromosome where they occurred, thus expanding the region without recombination between mating‐type chromosomes (Fig. 8b)
*Positive selection fixing inversions/rearrangements in the flanking region of the mating‐type locus:* the rearrangement breakpoints may in some cases induce beneficial genetic changes that can be selected for other causes than related to self‐recognition, but can only be fixed in the mating‐type chromosome where they occurred; beneficial introgressions also may be selected for in mating‐type chromosomes to counteract degeneration, possibly bringing rearrangements from another species; there also may be positive selection for rearrangements preventing recombination without direct selection for being associated to the mating‐type locus (e.g. in the case of spore killer/antidote systems), but linkage to the mating‐type locus could occur by chance or selection for recombination suppression could be easier in the margin of a region already without recombination. *Relaxed selection allowing for transposable element accumulation together with their silencing marks:* transposable elements (TEs) may accumulate in the flanking regions of the mating‐type locus in cases where it already shows a large region of suppressed recombination where TEs accumulate and spread nearby because they are partially sheltered or actively disperse near their mother copies (Fig. 8c); this can promote expansion of recombination suppression if their silencing marks prevent crossing‐overs.
**Proximate causes**
*Recombination modifiers:* localized genes or genetic elements controlling recombination rates, in *cis* or *trans*.
*Inversions/rearrangements*: the lack of collinearity between mating‐type chromosome impairs the occurrence of crossing‐overs.
*Chromatin or other epigenetic modifications* that impair the occurrence of crossing‐overs.

In basidiomycete fungi, normally each mating type produces and receives specific pheromones (Fig. 2c), and the PR mating‐type locus itself includes linked pheromone and pheromone receptor genes, where recombination between them stopped hundreds of millions of years ago. These basidiomycete mating‐type loci thus display the most ancient trans‐specific polymorphism known to date; the genes are only alignable between mating types as protein sequences (Devier *et al*., 2009). Also essential to successful mating in basidiomycete fungi, the mating‐type homeodomain (HD) locus consists of paired genes that together function in determining compatibility after syngamy. The protein products of the adjacent HD1 and HD2 genes dimerize between mating types, signaling zygotic development (Fig. 2c; see also Box 2) (Feldbrugge *et al*., 2004). Linkage of the HD1 and HD2 homeodomain genes to each other also ensures proper mating‐type function at this second locus, located on a different chromosome than the PR locus in most basidiomycetes (Feldbrugge *et al*., 2004; Butler, 2007; Nieuwenhuis *et al*., 2013). Both the PR and HD mating‐type loci thus constitute ‘supergenes’ *–* loci transmitted as a single unit but composed of several genes (Schwander *et al*., 2014). Some additional genes may have been incorporated into the mating‐type locus for proper nonself‐recognition; for example, in *Cryptococcus neoformans* it was shown that mating‐type specific alleles of ribosomal proteins were not interchangeable, being likely involved in promoting inheritance of nuclei of both mating types (Ianiri & Fang, 2020). Some basidiomycetes have multiple copies of the same pheromone alleles at their mating‐type locus, which need to be linked to each other and to the pheromone receptor gene for proper nonself‐recognition; such a situation, especially where duplication numbers differ between mating types (e.g. Kues, 2015; Xu *et al*., 2016), could be investigated as a potential case of mating‐type antagonism as these multiple copies seem to increase pheromone production and, thus, attractiveness more than just functioning as recognition, for which a single gene copy is sufficient (Nieuwenhuis & Aanen, 2012).

Box 2 **Why is there a post‐mating check point in basidiomycete fungi?**

Haploid cells in basidiomycetes with different alleles at the PR locus can undergo syngamy for mating, but abort rapidly if they carry the same allele at the HD locus (Kues, 2000). Such post‐mating suicide may seem costly from an evolutionary point of view, as it is a waste of the energy invested thus far in mating and may prevent the gametes involved to produce any progeny at all. However, post‐mating suicide may not be that costly in fungi. Indeed, mating‐type determinism by two loci, one involved in post‐mating compatibility, is restricted to basidiomycete fungi, including mushrooms, in which mating occurs mostly by mating between hyphae*. In mushrooms, the HD and PR alleles often display dozens or hundreds of alleles, thus finely informing about relatedness. Stopping the mating process after syngamy* can avoid investing resources in diploid selfing progeny, and without any cost actually beyond the energetic cost of syngamy*. The other hyphae of the mycelia then can still mate later with other, less related individuals. In the cases of other basidiomycetes, such as rusts and smuts, in which mating occurs between single‐celled gametes, multiple gametes from the same diploid individual often are produced locally; suicide after suboptimal syngamy* could preserve resources for the progeny of locally present siblings, thus being beneficial via kin selection (Gibson *et al*., 2012). Another explanation may be that maintaining the HD locus as an incompatibility locus is costly, but it cannot be lost because it is needed as a ‘developmental switch’ (Perrin, 2012).

## III. Incorporation at mating‐type loci of genes without function in mating compatibility but involved in mating, without clear benefit of linkage

In some fungi, recombination suppression proximal to the mating‐type loci incorporates additional genes not directly involved in mating compatibility (Coppin *et al*., 1997; Feldbrugge *et al*., 2004; Debuchy & Turgeon, 2006; Butler, 2007) (Fig. 2a,b; Table 1). In the ascomycete Sordariomycete dung fungus *Podospora anserina*, a single gene constitutes one mating‐type idiomorph whereas three genes constitute the alternative mating type, one being involved in subsequent stages of sexual development (Coppin *et al*., 1997). In sordariomycetes, the SMR1 gene (Mat1‐1‐2; Dyer *et al*., 2016) involved in the fructification (ascocarp) formation is in linkage with one of the mating‐type idiomorphs and is lacking in the other (Wilken *et al*., 2017; Wilson *et al*., 2019). However, experiments have shown that this gene functions as well anywhere else in the genome; it does not need to be linked to the mating‐type locus* (Arnaise *et al*., 2001; Dyer *et al*., 2016). In basidiomycetes, too, there are clades in which genes involved in mating processes other than the key mating‐type determinants are present at the mating type loci, such as protein kinases and transcription factors STE11, STE12 and STE20 in the Tremellales (Sun *et al*., 2019). In *Cryptococcus* spp. genes of the MAP kinase pathway involved in mating seem to have been anciently linked to the PR mating‐type locus* (Fraser *et al*., 2004; Fraser & Heitman, 2004). However, it remains unclear why it would be evolutionarily beneficial to link these genes involved in the physiological process of mating to a mating‐type locus* determining genetic compatibility. Some hypotheses would be that: (1) it helps the co‐regulation of these genes, as in gene clusters (Lawler *et al*., 2013) (e.g. the gene in one mating type is under control of a transcription factor in the other mating type, thus ensuring that expression will only occur in the diploid/dikaryotic phase) (Perrin, 2012); or (2) different alleles are optimal in the two mating types (i.e. mating‐type antagonistic selection*). There is little evidence so far of any benefit for linkage of most of these genes to the mating‐type locus* and some have been moved experimentally without any deleterious effect (Arnaise *et al*., 1997; Graïa *et al*., 2000; Contamine *et al*., 2004; Lambou *et al*., 2008; Grognet *et al*., 2019). It may thus be that most genes have been linked to the mating‐type locus by other evolutionary process, as outlined below and in Table 1, like many other genes not involved in mating but linked to the mating‐type locus*. This evolutionary question has been poorly recognized and therefore little studied.

## IV. Uniparental mitochondrion inheritance or selfing mating systems can trigger beneficial recombination suppression around fungal mating‐type loci

In some fungal mating‐type chromosomes*, recombination suppression has extended across chromosomal regions beyond linking together the mating‐type genes, thus enlarging the mating‐type ‘supergenes’ to include many more functions (Table 1). In basidiomycete fungi in the genus *Ustilago*, for example, two genes (*Iga2* and *Rga2*) involved in uniparental inheritance of mitochondria (Fedler *et al*., 2009) have been incorporated at the mating‐type locus* (Bortfeld *et al*., 2004); mitochondria are transmitted by a single mating type in many fungi (Basse, 2010; Sun *et al*., 2020), which most often is due to the active process of degradation of mitochondria from one mating type, thus avoiding heteroplasmy that may favor selfish mitochondria (i.e. being less efficient at respiration but replicating faster; Billiard *et al*., 2011). Likewise, the mitochondrial intermediate peptidase gene *mip* is linked to the HD locus in many basidiomycetes (James *et al*., 2004; Fraser *et al*., 2007; van Diepen *et al*., 2013); although this gene has no function in the mating process, it is involved in cleaving proteins in the mitochondrion, with mutants at this gene having degraded mitochondria (Isaya *et al*., 1995). Although uniparental inheritance of mitochondria can be beneficial for the nuclear genome as it minimizes the potential for the spread of selfish mitochondria haplotypes (Havird *et al*., 2019), selection for transmission of mitochondria by a single mating type thus does not constitute a mating‐type antagonism *per se* (it does not improve alternative mating‐type functions). Instead, it corresponds to a benefit to the nuclear genome as a whole of having uniparental inheritance (Cosmides & Tooby, 1981; Greiner *et al*., 2015).

In several selfing basidiomycetes, recombination suppression further links the HD and PR mating‐type loci (Bakkeren & Kronstad, 1994; Fraser & Heitman, 2005; Nieuwenhuis *et al*., 2013; Branco *et al*., 2017, 2018) (Fig. 2a; Table 1), which increases the odds of compatibility among the gametes of a diploid individual (Nieuwenhuis *et al*., 2013; Branco *et al*., 2017). Indeed, with linked HD and PR loci, a given diploid individual produces only two mating types among its progeny, instead of four as occurs with unlinked mating‐type loci; a given gamete is therefore compatible with half of other gametes instead of one quarter of them. The linkage of the two mating‐type loci thus is beneficial under selfing, and has occurred repeatedly in several basidiomycete genera, such as the *Microbotryum* anther‐smuts (Branco *et al*., 2017), the *Cryptococcus* human pathogen (Fraser & Heitman, 2005), the *Malassezia* human pathogens (Xu *et al*., 2007; Gioti *et al*., 2013), and the *Sporisorium* and *Ustilago* cereal smuts (Bakkeren & Kronstad, 1994; Que *et al*., 2014; Taniguti *et al*., 2015; Rabe *et al*., 2016; Liang *et al*., 2019). The region of suppressed recombination linking HD and PR loci can span from *c*. 100 kb and 20 genes in *C. neoformans* to 600 genes and megabases of DNA in *Microbotryum* fungi (Fraser & Heitman, 2005; Branco *et al*., 2017), trapping in‐between many genes with no functions in mating. These particular fungi are all pathogens, for which selfing may constitute reproductive insurance. Indeed, for pathogens mating within their host or just before infecting a host, a very limited number of genotypes may be present in or on the host. Beyond the advantage of reproductive assurance, selfing also can be selected for due to the automatic fitness advantage of transmitting twice as many of gene copies (Busch & Delph, 2012). In at least three genera or families – *Microbotryum*, *Ustilago, Cryptococcus –* multiple independent events of HD–PR linkage even occurred within clades (Bakkeren & Kronstad, 1994; Rabe *et al*., 2016; Branco *et al*., 2018; Liang *et al*., 2019; Sun *et al*., 2019), revealing frequent evolutionarily convergent events within each of these genera.

In some other selfing basidiomycete fungi, recombination suppression does not link mating‐type loci one to each other but does link the mating‐type determining loci to centromeres (Carpentier *et al*., 2019), which yields a similar elevated gamete compatibility odds under intra‐tetrad mating (i.e. automixis*) or mating among the products of a given meiosis. Indeed, if there is no recombination between the mating‐type loci and their respective centromeres, the mating‐type alleles segregate at the first meiotic division at both loci, and only two mating‐type genotypes are generated among the products of the meiosis (Carpentier *et al*., 2019).

**Fig. 3:**
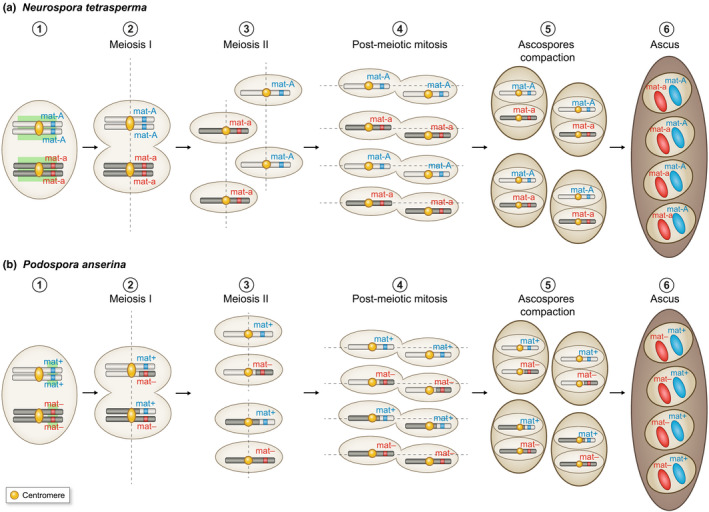
Illustration of the role of recombination suppression around the mating‐type locus in the ascomycetes *Neurospora tetrasperma* (a) and *Podospora anserina*(b) in compacting nuclei of opposite mating types in a spore. (1) Nuclei of the diploid cell before meiosis, heterozygous at the mating‐type locus. (2) First division of meiosis. No crossing‐over occurs between the mating‐type locus and the centromere in *N. tetrasperma* (a) and a single crossing‐over systematically occurs between the mating‐type locus and the centromere in *P. anserina* (b). (3) Second division of meiosis; in *N. tetrasperma,*the spindles are parallel and partially overlapping, which leads to the separation of the two sister nuclei. (4) Post‐meiotic mitosis. (5) Ascospore formation encompassingtwo nonsister nuclei from post‐meiotic mitoses in *N. tetrasperma* and *P. anserina* (the nuclei compacted in a spore being sister nuclei from the second division of meiosis in *P. anserina* and non‐sister nuclei from the second division of meiosis in *N. tetrasperma)*. (6) Heterokaryotic ascospores location in ascus (i.e. with two unfused nuclei). Only the mating‐type chromosomes are drawn with gray and black rectangles. The opposite mating‐type alleles (*mat*A/ *mat* a and *mat*+/*mat*‐ in *N. tetrasperma* and *P. anserina*, respectively) are represented by blue and red rectangles, respectively. The centromere location is represented by yellow rectangles. The region of recombination suppression on the mating‐type chromosome is shown on panel (1) by a green area. Spindle orientation in meioses and mitoses is indicated by dotted line.

Recombination suppression around the mating‐type locus in some fungi also can ensure that two nuclei of opposite mating types are placed together by cytological means in a single dispersing spore* (Fig. 3); this process of pseudo‐homothallism* allows the completion of the life cycle without the necessity of locating a haploid mating partner or to be universally compatible for mating (Billiard *et al*., 2011, 2012; Grognet *et al*., 2014; Grognet & Silar, 2015). In fungi belonging to the *Neurospora tetrasperma* species complex, which display such cytological post‐meiotic nuclear packaging, a large region of suppression of recombination (*c*. 7 Mb), spanning over more than 75% of the mating‐type chromosome, links the centromere and the mating‐type locus* (Gallegos *et al*., 2000; Menkis *et al*., 2008; Ellison *et al*., 2011; Corcoran *et al*., 2016). In *P. anserina* the occurrence of a single crossing‐over between the mating‐type loci and the centromere allows proper nuclear packaging with two opposite mating types per spore*. A 0.8‐Mb region of recombination suppression around the mating‐type loci likely plays a role in the limitation to a single crossing‐over in the relatively large region between the mating‐type locus* and the centromere (Grognet *et al*., 2014; Grognet & Silar, 2015).

## V. Further recombination suppression beyond fungal mating‐type loci without obvious benefits

**Fig. 4:**
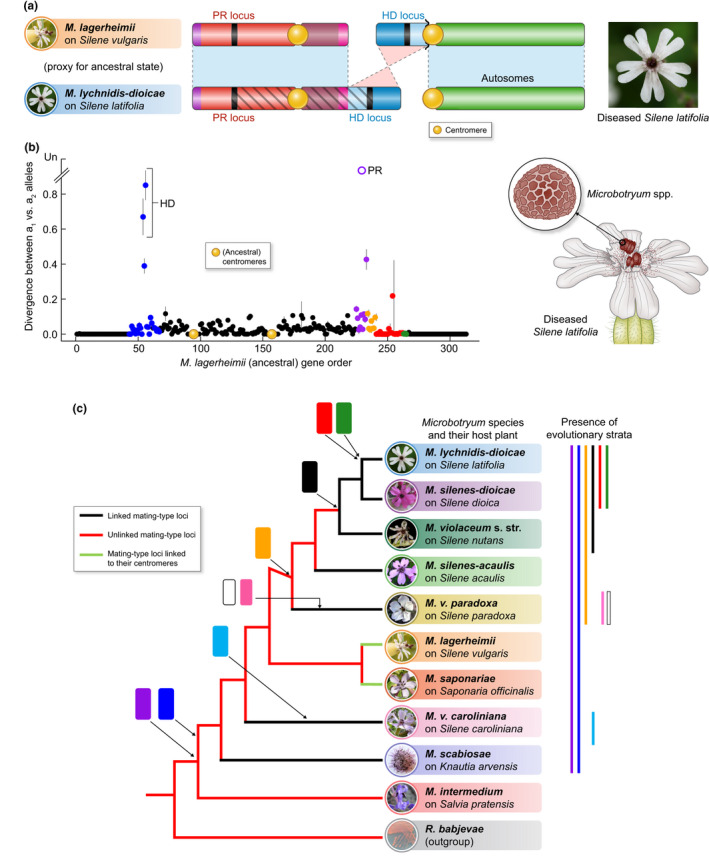
Independent evolution and stepwise extension of recombination suppression in the mating‐type chromosomes in the highly selfing plant castrating fungus *Microbotryum lychnidis‐dioicae* (Basidiomycete). (a) Rearrangements having led, from the ancestral state of unlinked mating‐type loci, to a single mating‐type chromosome and mating‐type locus linkage. A picture is shown of a diseased *Silene latifolia* flower with anthers full of fungal spores. (b) Evolutionary strata in *M. lychnidis‐dioicae*, as revealed by plotting the synonymous divergence (dS) between the alleles associated with the two mating types (a_1_ and a_2_) against ancestral gene order. Homeodomain (HD) and pheromone‐receptor (PR) are the two mating‐type loci, linked together by the black evolutionary stratum. Other evolutionary strata are shown in color, linking mating‐type genes with many other genes having functions unrelated to mating‐type determination, in several successive steps (Branco *et al*., 2017). The blue, purple and orange strata evolved around the mating‐type loci before PR–HD linkage, whereas the orange, red and green strata evolved successively after PR–HD linkage. When the divergence between *a1* and *a2* alleles was too extensive to be calculated, it was depicted as « unalignable» (Un).A drawing is shown of a diseased *Silene latifolia* flower with anthers full of fungal spores, with a close‐up on a spore in an inset.(c) Phylogeny of 10 *Microbotryum* species based on genome data (Branco *et al*., 2018), with red branches for unlinked HD and PR mating‐type loci, black branches for linked HD and PR loci, and green branches for mating‐type loci linked to their respective centromeres. The presence of the various evolutionary strata in the various species is shown on the right as well as the nodes where the various strata have been inferred to have arisen; evolutionary strata have the same colors as used in previous studies (Branco *et al*., 2017, 2018). The blue, purple and orange strata evolved as recombination suppression extension distal to the PR or HD loci when they were still on different chromosomes. The multiple events of HD–PR linkage then occurred independently in several lineages (black branches), involving different chromosomal arms and/or orientation of chromosomal fusions (Branco *et al*., 2017, 2018). For each *Microbotryum* species pictures of diseased flowers and name of the host plant are indicated. A red yeast species was used as outgroup (*Rhodosporidium babjevae*).

Further extension of recombination suppression has been reported around mating‐type loci to genes without any function related to mating, mating types, mating‐type compatibility or mitochondrion inheritance. Recent studies of mating‐type chromosomes* in fungi without male*/female* roles (Branco *et al*., 2017) (the anther‐smut, plant‐castrating *Microbotryum* fungi) have shown that evolutionary strata have evolved repeatedly in the absence of sexual antagonism*. Multiple evolutionary strata extended the region without recombination, in several steps, beyond mating‐type genes and centromeres, into regions devoid of genes involved in mating‐type determination. This expansion has occurred independently in several *Microbotryum* lineages (Branco *et al*., 2018) (Fig. 4; Box 3). The nonrecombining region can extend up to 90% of the chromosome, which is nearly 4 Mb long (Hood *et al*., 2013; Badouin *et al*., 2015; Branco *et al*., 2018). Because neither mating‐type chromosome recombines, they both accumulate rearrangements independently (Bull, 1978), and it is not possible to observe ancestral gene order directly for assessing evolutionary strata (whereas the X sex chromosome can be used to recover the ancestral‐like order as it recombines in females; Fig. 1). In the fungal system, the ancestral gene order could be inferred from the gene order shared by several distant *Microbotryum* species that have retained recombining mating‐type chromosomes* until recently, where recombination preserves gene order (Branco *et al*., 2017). Seven independent cases of evolutionary strata were identified across the *Microbotryum* phylogeny (Fig. 4; Box 3), involving different sets of genes and devoid of mating‐type genes (Branco *et al*., 2017, 2018). The repeated evolution of such strata in closely related *Microbotryum* anther‐smut species suggests that they evolve frequently in response to common evolutionary processes.

Box 3 **Historical review of discoveries related to mating system, mating types and recombination suppression in *Microbotryum* fungi**

*Microbotryum violaceum* (*sensu lato*) causes anther‐smut disease in plants of the Caryophyllaceae family. These fungi are important research models in many fields of biology, including genomics, host–pathogen interactions and evolutionary ecology. In the early work on sexual compatibility, *Microbotryum violaceum*, called *Ustilago violacea* before 1982, was among the first fungi in which heterothallism* was demonstrated (Kniep, 1919).

More recently, the mating‐type chromosomes* of *M. lychnidis‐dioicae* were the first to be found size‐dimorphic in fungi, as initially revealed by electrophoretic karyotypes, and with extensive nonrecombining regions, as supported by AFLP markers, being thus suggested to share genomic features with sex chromosomes* (Hood, 2002; Hood *et al*., 2004). A later study suggested that the nonrecombining region on the *M. lychnidis‐dioicae* mating‐type chromosomes* was small (*c*. 1000 kb) but the size estimate was based on a few markers (Votintseva & Filatov, 2009). An optical map confirmed that the region of recombination suppression spanned 90% of the mating‐type chromosomes* (Hood *et al*., 2013). Votintseva & Filatov (2009) also claimed the existence of evolutionary strata based on heterogeneous synonymous divergence values in the nonrecombining region, but no physical order of the genes analyzed had been provided, the variation in the degree of the allelic divergence did not look like discrete strata, and only few genes were analyzed. A subsequent study reported an absence of correlation between the dS level from the study by Votintseva & Filatov (2009) and the age of the linkage of each marker to the mating type estimated based on gene genealogies (Petit *et al*., 2012). Another study claimed to have found evolutionary strata on the *M. lychnidis‐dioicae* mating‐type chromosomes* based on a clustering method (Pandey & Azad, 2016), but later studies showed the inferred strata were not correct, as the current gene order results from chaos of rearrangements (see below).

The sequencing of cDNA libraries allowed the identification of the pheromone receptor (PR) gene (Yockteng *et al*., 2007) and later of the HD genes (Petit *et al*., 2012). STE3‐like pheromone receptors have been characterized among sequences expressed during mating of *M. lychnidis‐dioicae* (Yockteng *et al*., 2007). Gene genealogies of the alternate alleles of the pheromone receptor gene from multiple basidiomycete species revealed in *Microbotryum* the deepest degree of trans‐specific polymorphism ever reported. The *Microbotryum* a_1_ and a_2_ pheromone receptor alleles had been estimated to diverge 370 Myr ago (Devier *et al*., 2009). The HD genes in *Microbotryum* were identified in the ESTs (Petit *et al*., 2012) by sequence similarity with the previously characterized homeodomain in *Sporobolomyces* spp. (Coelho *et al*., 2010). Incomplete trans‐specific polymorphism signal has been found in HD gene trees with several *Microbotryum* species. Indeed, HD alleles from a_1_ and a_2_ strains clustered by species in some groups and clustered by mating type with certain level of trans‐specific polymorphism in other groups. The recombination suppression at the HD genes could still be ancestral in the *Microbotryum* clade, with some rare crossing‐overs or gene conversion events that have reset the allelic divergence in some groups (Petit *et al*., 2012).

Pheromone genes have been identified in *Microbotryum* spp. by sequence similarity with pheromone genes described in the Microbotryomycete *Rhodosporidium toruloides* fungus and their mating function has been validated experimentally (Xu *et al*., 2016). Studying pheromone genes and pheromone receptor gene sequences from a_1_ and a_2_ mating types of multiple *Microbotryum* species suggested the existence of distinct coevolutionary patterns between the two pairs of pheromones – pheromone receptors. The a_1_ pheromone allele is present in many copies within the mating‐type locus* and shows little variation across *Microbotryum* species. This low variation is matched by its corresponding a_2_ pheromone receptor gene. By contrast, the a_2_ pheromone allele is present in few copies within a genome and is more diverse in sequence across species than the a_1_ pheromone. These differences in coding sequence variation across *Microbotryum* species were associated with differences in the strength of purifying selection, which was stronger on the a_1_ pheromone allele. These results are consistent with earlier observations on *Microbotryum* mating behaviour (Day, 1976): a_1_ cells initiate mating through greater diffusion of a conserved a_1_ signal, whereas the a_2_ cells play a responsive role through a_2_ pheromones which do not need to be as conserved as the a_1_ signal. The conjugation tube following the pheromone recognition develops more rapidly and to a greater extent from a_2_ than from a_1_ cells.

Recently, long‐read sequencing and high‐quality assembly confirmed the existence of a large region of recombination suppression in *M. lychnidis‐dioicae* as seen in chromosomal optical maps (Hood *et al*., 2013), spanning around 90% of the mating‐type chromosome length (i.e. > 3 Mb), with a chaos of structural rearrangements (Badouin *et al*., 2015) and elevated synonymous divergence values between a_1_‐ and a_2_‐associated alleles. Despite the heterogeneous and elevated synonymous divergence values reported, no pattern of progressive extension of the nonrecombining region had then been found because the high degree of rearrangements on the two mating‐type chromosomes* obscured the ancestral gene order (Badouin *et al*., 2015). This issue was resolved by using the gene order present across distant and related species (*M. intermedium* and *M. lagerheimii*) with high synteny at the mating‐type chromosomes*, allowing the identification of evolutionary strata (Fig. 4) (Branco *et al*., 2017).

**Fig. 5:**
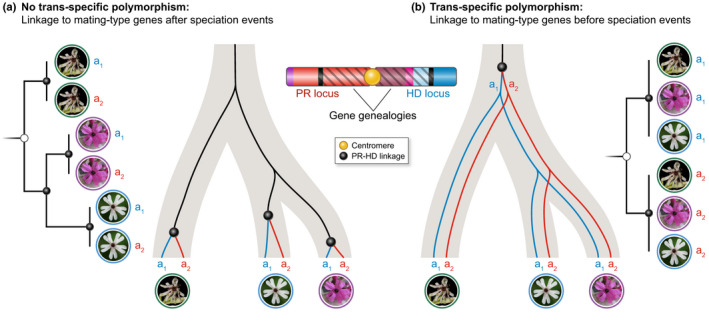
Illustration of trans‐specific polymorphism at a gene located between the Homeodomain (HD) and pheromone‐receptor (PR) loci and thus linked to mating‐type loci in *Microbotryum* mating‐type chromosomes, and its use to infer the relative age of linkage to mating‐type loci relative to speciation dates. If recombination suppression is younger than speciation, alleles associated to different mating types cluster per species (a), whereas if recombination suppression is older than speciation, alleles associated to different mating types cluster together across species (b).

Segregation analyses in progenies had shown that although many *Microbotryum* species carried linked HD and PR loci, some still had HD and PR loci on different chromosomes (*M. intermedium, M. saponariae* and *M. lagerheimii*; Fig. 4c) (Hood *et al*., 2015). Given the *Microbotryum* phylogeny and the distribution of species with linked mating‐type loci (Fig. 4c), the most parsimonious hypothesis was an ancestral event linking HD and PR loci, followed by a reversal to an unlinked state (Hood *et al*., 2015). However, comparative genomic analyses revealed several independent and convergent events (Branco *et al*., 2018) rather than one ancient linkage event followed by a reversal: the linkage between HD and PR indeed occurred through different rearrangements and chromosomal fusions in the different species (Branco *et al*., 2018), involving different chromosomal arms and/or orientation of fusion (different black branches in Fig. 4c). The independent evolution of recombination suppression was further checked based on the age of trans‐specific polymorphism (Fig. 5). Furthermore, dS patterns on mating‐type chromosomes plotted along the ancestral gene order in each species revealed multiple independent stepwise extension (Branco *et al*., 2018) (Fig. 4c), with multiple evolutionary strata of various ages across the phylogeny (strata of different colors in Fig. 4c).

Regarding species with HD and PR loci on different chromosomes (*M. saponariae* and *M. lagerheimii*; Fig. 4c), progeny segregation analyses have shown that the two mating‐type loci were linked to their respective centromeres (green branches in Fig. 4c; Hood *et al*., 2015; Carpentier *et al*., 2019) and genomic analyses revealed that the linkage likely occurred convergently as independent events (Carpentier *et al*., 2019).

Although the challenge of reconstructing ancestral gene order in mating‐type chromosomes* can be overcome, other mutational considerations are involved in quantifying evolutionary strata in fungi. In *Microbotryum* fungi, several lines of evidence have been provided for establishing the existence of evolutionary strata (Box 3). The synonymous divergence (dS) between alleles on the two mating‐type chromosomes* was plotted along the ancestral gene order in several species, and the shared presence of stretches of likewise elevated divergence in a given span of chromosome was used to infer the phylogenetic node at which recombination ceased in that genomic region (Fig. 5). The inference of the different strata was then further validated by testing whether the dS level was significantly different among the different strata within each species. A third line of evidence used to support the inferred node of appearance of the various evolutionary strata was based on the deepness of trans‐specific polymorphism at the genes in evolutionary strata. This approach utilizes the principle that, as soon as recombination cessation links a gene to the mating‐type locus*, the alleles at this gene will accumulate differentiating mutations in the genomes of alternative mating types. If a gene becomes linked to the mating‐type loci before a speciation event, the genealogy of this gene will group together the alleles of the same mating type across species (Fig. 5), which is called trans‐specific polymorphism. Going deeper in the phylogeny, the nodes where alleles cease to cluster according to mating type, and rather cluster according to species, thus provide indication on the time since recombination suppression (Fig. 5). However, gene conversion events, even if very rare, will reset the level of trans‐specific polymorphism (Sun *et al*., 2012). As a matter of fact, the level of trans‐specific polymorphism often was not homogeneous within evolutionary strata in *Microbotryum*, but the oldest trans‐specific polymorphism within a stratum can give strong support to the stratum's inferred age. As an extreme case, an old mating‐type loci linkage has been inferred in the Tremellales based on shared gene order and rearrangements across species, and yet there was no trans‐specific polymorphism, which was interpreted as resulting from the occurrence of rare gene conversion events (Sun *et al*., 2019).

Recombination suppression also has been shown to extend around the mating‐type locus* in the button mushroom *Agaricus bisporus* (Xu *et al*., 1993). The different varieties of the button mushroom have different breeding systems, either homothallic*, pseudo‐homothallic* or heterothallic*. The cultivated *A. bisporus* var. *bisporus* variety is pseudo‐homothallic, reproduces mainly by automixis* and has very extensively suppressed recombination (Sonnenberg *et al*., 2016), across almost the whole genome, with crossing‐overs only occurring at the very ends of all chromosomes (Foulongne‐Oriol *et al*., 2009, 2010, 2016; Morin *et al*., 2012; Sonnenberg *et al*., 2016, 2017). Conversely, the variety *A. bisporus* var.* burnettii* is heterothallic and exhibits a quite homogenous recombination pattern along chromosomes, except in some genomic regions where recombination is low or suppressed, including the mating‐type locus region (Foulongne‐Oriol *et al*., 2010). Only the HD mating‐type locus* is involved in mating‐type determination in *A. bisporus*, even the variety *burnettii* with no large recombination suppression, so that recombination suppression is unlikely to have evolved under selection for linking mating‐type loci together (Morin *et al*., 2012). The recombination suppression may still have evolved to promote automixis* as in *Microbotryum, P. anserina* and *N. tetrasperma* fungi, for example by linking HD to the centromere. It is, however, unclear why the recombination suppression evolved and extended differently in several varieties of *A. bisporus*. Such extension may constitute evolutionary strata without any relationship to mating‐type genes. Another factor that may be relevant to the recombination landscape is that the BSN locus (basidial spore* number), determining the number of nuclei per dispersing spore* and thus the automictic* versus outcrossing behaviors, is linked to the mating‐type locus (Imbernon *et al*., 1996). Within the *Agaricus* genus, the recombination suppression around the mating‐type locus also has been reported in the distant species *A. subrufescens* (Thongklang *et al*., 2014; Foulongne‐Oriol *et al*., 2016) and *A. sinodeliciousus* (Ling *et al*., 2019). In *A. subrufescens*, a genetic linkage map suggested a normal recombination behavior on the rest of the genome (Foulongne‐Oriol *et al*., 2016).

**Fig. 6:**
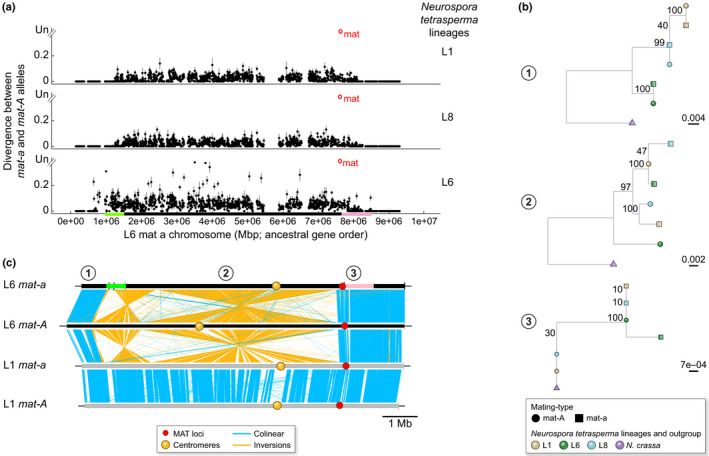
Evolutionary strata in the ascomycete *Neurospora tetrasperma* species complex. (a) Divergence (synonymous divergence, dS) values were plotted according to the gene order in the closely related species *Neurospora crassa* with recombining mating‐type chromosomes that likely represents a good proxy for the ancestral gene order. Dots indicate mean values and bars standard deviation of dS per gene. When the divergence between alleles was too extensive to be calculated, it was depicted as «unalignable» (Un). Pink and green colors on the dS plot illustrate putative evolutionary strata. (b) Maximum‐likelihood trees of single genes located in (1) the pseudo‐autosomal region (gene *mRNA_2374*), (2) the black oldest evolutionary stratum (gene *mRNA_2079*) and (3) the more recent pink evolutionary stratum (gene *mRNA_2177*). Bootstrap values (out of 100 replicates) are indicated at nodes. Trans‐lineage polymorphism is present in the black and pink evolutionary stratum. dS values and tree per genes were computed as described previously (Branco *et al*., 2017). *Mat‐A* and *mat‐a* haploid genomes of the dikaryotic* P4495, J13 and P581 strains belonging to the L1, L8 and L6 lineages of the *N. tetrasperma* species complex (respectively) were retrieved from Corcoran *et al*. (2016) and gene annotation of the L6 *mat‐a* genome was used. For trees, *N. crassa* was used as outgroup and genome was retrieved from Ellison *et al*. (2011). (c) Synteny comparison between the L6*mat‐a* and L6*mat‐A* mating‐type chromosomes of the dikaryotic* P581 strain that shows large rearrangements (Ellison *et al*., 2011) and between the L1 *mat‐a* and L1 *mat‐A* mating‐type chromosomes of the dikaryotic* P4492 strain that shows no rearrangements (Y.Sun *et al*.,^2017^). Mating‐type locus (mat) locations are represented by red circles. Centromere location is represented by yellow rectangles.

**Fig. 7:**
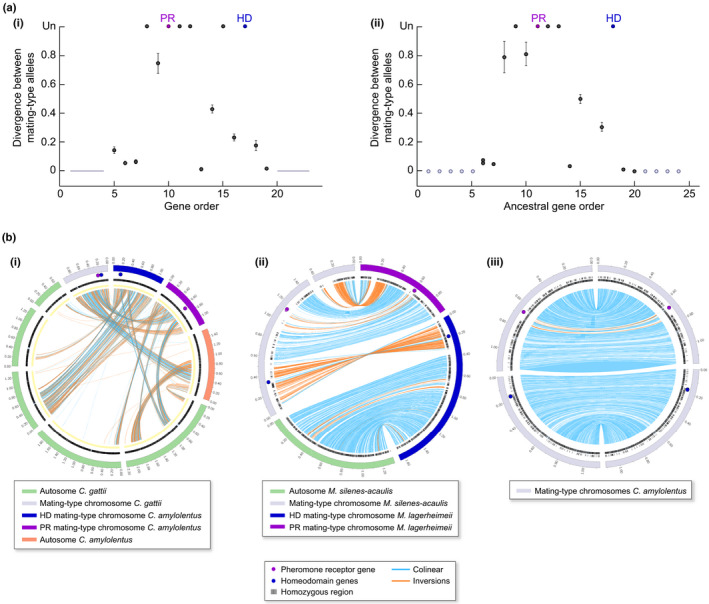
Evolutionary strata in the basidiomycete *Cryptococcus* species. (a) Divergence (synonymous divergence, dS) values between *a* and *alpha* mating‐type associated alleles along the mating‐type chromosomes within diploid individuals of two*Cryptococcus species*: (i) *C. neoformans alpha* JEC21 strain and *a* JEC20 strain (the whole region sequenced in the *a* JEC20 strain in black; gray lines correspond to values inferred from the isogenic state between the two strains (Hull *et al*., 2005), and not based on genomic data); (ii) *C. gattii alpha* WM276 and *a* E566 strains dS was plotted following the methods in (Branco *et al*., 2017) according to the gene order in *C. amylolentus* (strain CBS6039) as a proxy for ancestral gene order, as it is a closely related species with unlinked mating‐type loci and recombining mating‐type chromosomes (S.Sun *et al*., 2017). Pheromone receptor (PR) and homeodomain (HD) genes are indicated in purple and blue, respectively. Only genes currently between HD and PR genes, or having nonzero dS values and being close to PR and HD genes, have been plotted between HD and PR, not those ancestrally between HD and PR and that moved to pseudo‐autosomal regions with dS = 0. (b) Comparison of gene orders between *Cryptococcus* fungi with linked HD and PR mating‐type loci with their ancestral‐like gene order inferred from closely related species with HD and PR loci on separate chromosomes. Syntenic regions are indicated by blue links and inversions by orange links. Zero dS values are indicated by black traits on the inner tracks and centromeric repeats by yellow traits. Comparisons shown are (i) between the *Cryptococcus gattii alpha* WM276 strain and the *C. amylolentus* CBS6039 strain as a proxy for ancestral gene order; (ii) between the *Microbotryum silenes‐acaulis* and *M. lagerheimeii* as a proxy for ancestral gene order; and (iii) between the two mating‐type chromosomes of the *C. amylolentus* CBS6039 strain.

The existence of evolutionary strata extending recombination suppression beyond mating‐type loci and beyond linkage to the centromere also has been suggested in the fungi belonging to the *N. tetrasperma* species complex (Menkis *et al*., 2008), as well as in *Cryptococcus neoformans* and in *C. gattii* (Fraser *et al*., 2004), although no divergence plots along ancestral gene order have been published and introgressions detected in some *N. tetrasperma* lineages could have led to spurious stratum patterns. We therefore plotted herein divergence between mating types along an ancestral order inferred from recombining outgroups in *N. tetrasperma* lineages without introgressions (L1 and L8; Corcoran *et al*., 2016)) and indeed found evidence of evolutionary strata (Box 4; Figs 6a, 7a). Evolutionary strata also have been found in *Podospora pseudocomata* (FEH, PS, TG, unpublished data).

Box 4 **Evolutionary strata in *Neurospora tetrasperma* and *Cryptococcus* fungi**

We plotted the divergence between mating types for strains of the L1, L6 and L8 lineages of the *N. tetrasperma* cryptic species complex along an inferred ancestral gene order of the mating‐type chromosome *–* the gene order of the closely related fungal species *N. crassa* with recombining mating‐type chromosomes* (Ellison *et al*., 2011; Y. Sun *et al*., 2017). We confirmed the existence of a region of significantly lower divergence in the right flanking region of the mating‐type locus compared to the left flanking region, that likely constitutes a recent evolutionary stratum (in pink in Fig. 6a), as suggested previously (Menkis *et al*., 2008). We also found this pink recent stratum in the L1 and L8 lineages of *N. tetrasperma* (Fig. 6a), indicating that it was not the spurious result of the introgression detected in the L6 *N. tetrasperma* lineage (Corcoran *et al*., 2016). The dS was non‐null in this region (i.e. there was some heterozygosity), whereas all autosomes in the L6 and L8 lineages were completely homozygous (not shown). We found trans‐lineage polymorphism in this pink stratum (Fig. 6b) that can be the result of introgression in L6, but not in L1 or L8, supporting a recombination suppression having occurred before the divergence of the lineages. Furthermore, the dS plots suggest the existence of another stratum, present only in the L6 lineage, in the left flanking region (in green in Fig. 6a). An inversion in the L6 lineage encompasses the green stratum, supporting the occurrence of recombination suppression (Fig. 6c). This stratum might be due to introgression (Corcoran *et al*., 2016), but nevertheless represent an extension of recombination suppression, as shown by the inversion.

We also plotted the divergence between mating types in *C. neoformans* and in *C. gattii* (Fig. 7a), along an inferred ancestral gene order of the mating‐type chromosome *–* the gene order of *C. amylolentus* that has unlinked mating‐type loci and recombining mating‐type chromosomes* (S. Sun *et al*., 2017). The plots of divergence between mating types (Fig. 7a) support the existence of evolutionary strata for the two species: older strata with very high divergence around each of the HD and PR mating‐type loci, and a younger stratum with moderate divergence in genes flanking the first strata and linking HD and PR loci, contrasting with null divergence in the recombining regions. Considering the *C. amylolentus* gene order as a proxy for the ancestral state, the rearrangements linking HD and PR loci in *C. gattii* (Fig. 7b) appear to have involved many more fusions and fissions with autosomes than in the anther‐smut *Microbotryum* fungi (Fig. 7b). Even in *C. amylolentus* with unlinked mating‐type loci and recombining mating‐type chromosomes, rearrangements can be observed around the PR mating type locus (Fig. 7b), supporting the existence of an ancient stratum here shared by various *Cryptococcus* species.

In many of the ascomycetes, the mating‐type locus is found in linkage with APN2 (encoding an endonuclease/DNA lyase) and SLA2 (encoding a protein binding to cortical patch actin) (Butler, 2007). A large region without recombination also has been reported near the mating‐type locus in European populations of the ascomycete pathogen *Cryphonectria parasitica* responsible for the chestnut blight (Kubisiak & Milgroom, 2006). Recent genome sequencing suggests the presence of rearrangements between strains around the mating‐type loci in several fungi. In the yeast *Lachancea kluyveri* recombination suppression has been reported across 1 Mb around the mating‐type locus, involving many genes not related to mating or mating‐type functions (Friedrich *et al*., 2015; Brion *et al*., 2017, 2020). The additional genes incorporated into linkage with the mating‐type locus sometimes involved genes that were not initially physically the most proximal to the mating‐type genes. For example, in the apple ascomycete pathogen *Valsa mali*, two genes (*COX13* and *APN2*) were linked to the mating‐type locus*, long after initial recombination suppression at mating‐type genes, via intrachromosomal rearrangements (Yin *et al*., 2017).

In basidiomycetes also, genes with no known function in mating have been incorporated into the mating‐type locus, such as the beta‐flaking gene (*bfg*) present next to the HD genes in many Agaricomycota (Rong *et al*., 2015). Recent inversions near the mating‐type locus* have been reported in the mushroom *Flammulina velutipes* (van Peer *et al*., 2011). Other examples of recombination suppression extending beyond mating‐type loci include *Ustilago bromivora* in which two evolutionary strata seem to exist, one on either side of the mating‐type locus* (Rabe *et al*., 2016).

In many other fungi, the lack of recombination is shown by the presence of genes close to mating‐type genes in one mating type and completely lacking in the other mating type (hemizygosity*). This situation indicates that a gene has been incorporated at the mating‐type locus* by recombination suppression, followed by the loss of the focal genes in linkage to one mating‐type allele; gene loss in one allele is allowed by the sheltering in a permanent heterozygote state in the region without recombination around the mating‐type locus*. Alternatively, there may have been gene gains in the mating‐type locus in a single mating type. This situation is analogous to the hemizygous* genes on the heterogametic* Y in mammals or *Drosophila* (Bachtrog, 2005). Across ascomycetes for example, six genes with no known function in mating have been found specific to one mating type and 10 other genes specific to the other mating type, with no homology between them (Wilken *et al*., 2017; Wilson *et al*., 2019). These mating‐type specific genes often are shared by whole clades, indicating ancient recombination suppression (Wilken *et al*., 2017; Wilson *et al*., 2019), but different genes have been incorporated at the mating‐type locus* in different fungal clades. In Leotiomycetes for example, a gene encoding a metallothionein protein without known role in mating is found in a single mating type (Wilken *et al*., 2017; Wilson *et al*., 2019). Other examples include the model fungi *Neurospora crassa, Magnaporthe oryzae, Coccidioides* spp., *Cordyceps* spp., *Fusarium* spp. and *Melampsora larici‐populina* (Persoons *et al*., 2014; Wilken *et al*., 2017; Wilson *et al*., 2019). The occurrence of various mating‐type specific (i.e. hemizygous*) genes, present in whole fungal clades but being different among clades, suggest multiple extension events of recombination suppression *–* evolutionary strata. Genomic analyses thus have recently revealed that the expansion of recombination around mating‐type loci may be a common and recurrent phenomenon across fungi, making their study in a comparative context potentially a powerful approach for gaining insights into the evolutionary genomics of sexual compatibility.

## VI. Consequences of recombination suppression: genomic degeneration

The lack of recombination typically leads to genomic degeneration. There have been several studies in fungi showing that nonrecombining regions on the mating‐type chromosomes* accumulate deleterious mutations, in terms of nonsynonymous substitutions, nonoptimal codon usage, accumulation of transposable elements, gene losses and decrease in gene expression level. Examples have been reported in *Microbotryum* fungi (Fontanillas *et al*., 2015; Branco *et al*., 2017; Bazzicalupo *et al*., 2019; Ma *et al*., 2020), in *U. hordei* (Bakkeren *et al*., 2006), in *P. anserina* (Grognet *et al*., 2014), in *N. tetrasperma* (Whittle & Johannesson, 2011; Whittle *et al*., 2011; Samils *et al*., 2013), and in the yeasts *L. kluyveri* (Brion *et al*., 2020) and *Saccharomyces cerevisiae* (de Clare *et al*., 2011). Experiments also have revealed haplo‐lethal alleles linked to mating‐type loci in several fungi, which are due to the existence of lethal alleles in nonrecombining regions near the mating‐type loci and have been reported in *Microbotryum* fungi (Oudemans *et al*., 1998; Thomas *et al*., 2003), *A. bisporus* (Callac *et al*., 2006), *Ustilago bromivora* (Rabe *et al*., 2016), *U. nigra* (Darlington & Kiesling, 1975), *U. bullata* (Fischer, 1940), *U. avenae* and *U. kolleri* (Grasso, 1955; Holton & Dietz, 1960).

Differential expression between alleles associated to different mating types often has been interpreted in terms of selection for different optima between mating types (Fraser *et al*., 2003; Samils *et al*., 2013; Grognet *et al*., 2014). However, such differential gene expression also can result from simple degeneration resulting from recombination suppression. In particular, differential expression between mating types was found associated with footprints of degeneration in the least expressed allele in *Microbotryum* fungi, such as transposable element insertions, indel distribution, and premature stop or nonsense codons (Ma *et al*., 2020). In turn, the accumulation of deleterious alleles and transposable elements could promote further extension of recombination suppression, as outlined below.

## VII. Ultimate and proximate mechanisms generating evolutionary strata beyond sexual antagonism or selection for mating‐type compatibility or mitochondrion inheritance

If evolutionary strata suppressing recombination far beyond the mating‐type genes regularly evolve in fungi, understanding why can reveal analogous causes that also might occur in other types of organisms. The most common hypothesis invoked for explaining evolutionary strata on sex chromosomes remains sexually antagonistic selection. Although the evolutionary strata on mating‐type chromosomes* provide a strong analogy to sex chromosomes* of animals and plants, it is important to emphasize the absence of any male*/female* roles associated to mating type, so sexual antagonism* cannot account for the observed extension of recombination suppression beyond mating‐type determining genes in fungi (see the Introduction section above). Mating type regulates haploid‐cell compatibility strictly through molecular signaling, promoting mating between different genotypes as does self‐incompatibility* in some hermaphroditic* plants. Few traits appear to have alternative forms beneficial in only one mating type beyond molecular signaling, rendering any kind of strong antagonistic selection* unlikely as a cause for evolutionary strata (Schafer *et al*., 2010; Bazzicalupo *et al*., 2019).

Furthermore, in the case of *Microbotryum* fungi, for example, all gametes have the same size (Schafer *et al*., 2010), and mating occurs quickly after meiosis, with no substantive free‐living haploid stage during which cells of opposite mating types might express contrasting ecological traits (Hood & Antonovics, 2000; Hood & Antonovics, 2004). A study on gene expression found no evidence of genes differentially expressed or with signs of sequence specialization between mating types in the young evolutionary strata in *Microbotryum* fungi, further reinforcing the view that there are not mating‐type antagonistic traits* (Bazzicalupo *et al*., 2019). Sexual antagonism*, or the analogous mating‐type antagonism cannot, therefore, account for the multiple evolutionary strata found in several *Microbotryum* species. The only functions known to be associated with mating types in *Microbotryum* fungi are the conjugation tube elongation and mitochondrion inheritance, and neither can explain the multiple recent evolutionary strata in *Microbotryum* fungi, trapping many different genes across the phylogeny.

More generally, evolutionary strata not involving any mating‐type genes thus seem to evolve regularly in fungi without sexual antagonism* and without being involved in mating‐type compatibility odds or mitochondrion inheritance. We therefore need to test other hypotheses to explain the spread of recombination suppression (Antonovics & Abrams, 2004; Ironside, 2010; Branco *et al*., 2017; Ponnikas *et al*., 2018). This effort can best proceed through combined attention to evolutionary and proximate explanations for recombination cessation (Table 1): evolutionary (ultimate) hypotheses explain *why*, in terms of evolutionary forces, chromosomal regions became linked to mating‐type loci (i.e. what gain in fitness it conferred or why neutral evolutionary processes are likely to act and lead to such situations); by contrast, proximate explanations focus on *how,* mechanistically, recombination was suppressed. These two aspects constitute two important, associated and interrelated, but very different levels of explanations, which, when combined, can produce a fuller understanding of the evolution of recombination suppression.

**Fig. 8:**
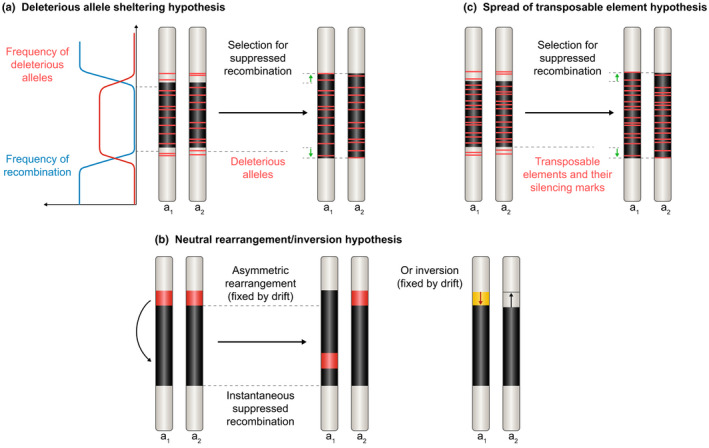
Three other evolutionary hypotheses than sexual antagonism for explaining evolutionary strata extending beyond mating‐type loci. (a) Sheltering of deleterious recessive alleles, which may accumulate in the recombining regions in linkage disequilibrium with the non recombining region on both sex and mating‐type chromosomes (here, the a_1_ and a_2_mating‐types). (b) Neutral rearrangements fixed by drift (the illustrated cases of gene movement and inversion seem to have occurred in *Microbotryum* fungi (Branco *et al*., 2017). (c) Spread of transposable elements and their silencing marks (methylation, heterochromatin, repeat‐induced point mutation) proximally to the region without recombination.

One evolutionary hypothesis is the successive linkage of a suite of genes with recessive deleterious mutations to the initial region of suppressed recombination (Antonovics & Abrams, 2004; Ironside, 2010; Branco *et al*., 2017). If recombination frequency gradually increases from the boundary of the nonrecombining region into the adjacent recombining region (the ‘deleterious allele sheltering hypothesis’; Fig. 8a; Table 1), then partial linkage (linkage disequilibrium) to mating‐type or sex‐determining genes may partially shelter them as the necessity to mate between different mating types preserves heterozygosity. This would lead to deleterious allele accumulation at the margin of the nonrecombining region. Occasional meiotic crossovers would generate individuals homozygous for deleterious alleles, reducing fitness through the exposure of genetic load. Thus, selection might favor the complete cessation of recombination (evolutionary cause) through recombination modifiers or rearrangements (proximal causes). Permanent sheltering may occur more readily than purging if recombination is rare relative to mutation accumulation near the region of suppressed recombination (Fig. 8a), although this remains to be tested, both theoretically and experimentally. Deleterious allele accumulation is known to occur in nonrecombining regions (Marais *et al*., 2008; Llaurens *et al*., 2009; Immler & Otto, 2015). However, deleterious allele accumulation may not necessarily be a simple consequence of a complete lack of recombination as modeled so far. Models have shown that deleterious alleles can accumulate under low rates of recombination (Grossen *et al*., 2012), but have yet to investigate whether such genetic load can drive recombination suppression, beyond the particular case of *Microbotryum* and high intra‐tetrad selfing rates (Antonovics & Abrams, 2004).

A second, nonexclusive hypothesis involves the fixation of rearrangements (Ironside, 2010; Ponnikas *et al*., 2018). Rearrangements, whether introduced in populations by mutations or introgressions, might be fixed by genetic drift or by positive selection for reasons other than the linkage to mating type that they cause. Inversions and asymmetric rearrangements in the flanking regions of nonrecombining loci can only be fixed in a single mating‐type chromosome and then automatically extend the nonrecombining region (Fig. 8b). Here rearrangements constitute proximate causes, and the ultimate explanation rests on whether they are fixed by drift or selection. Rearrangements that span the boundary of the nonrecombining region of one sex or mating‐type chromosome may indeed be neutral in the short term and become fixed on the sex or mating‐type chromosomes* where they arose as a result of linkage to mating‐type or sex‐determining genes (‘neutral rearrangement/inversion hypothesis’; Fig. 8b; Table 1). Rearrangements also can be selected for but for other reasons than being associated with the mating‐type locus. Rearrangements that distinguish alternative mating‐type chromosomes can be brought by introgression from another species, and be beneficial in nonrecombining mating‐type chromosomes by counteracting genomic degeneration (Corcoran *et al*., 2016; Hartmann *et al*., 2020). Rearrangements also could be beneficial by changing gene regulation (Fleiss *et al*., 2019) or if the breakpoint in itself induces an advantageous mutation or by suppressing recombination between a spore killer and its antidote (Svedberg *et al*., 2018). Actually, some genetic selfish elements have been found associated with fungal mating‐type loci (Meunier *et al*., 2018).

Some of the recent evolutionary strata identified in *Microbotryum* anther‐smut fungi involve inversions or movement of the region at the margin of the nonrecombining region into the middle of the nonrecombining region (Branco *et al*., 2017, 2018) (Fig. 8b). In the L10 lineage of *N. tetrasperma*, a large inversion appeared polymorphic (Y. Sun *et al*., 2017), extending the region of recombination suppression, which brings support to the hypothesis that neutral rearrangements fixed by drift may be a mechanism generating evolutionary strata. Examples also may include inversions found adjacent to the mating‐type locus in *Flammulina velutipes* (van Peer *et al*., 2011) and in several lineages of *N. tetrasperma* (Ellison *et al*., 2011; Y. Sun *et al*., 2017). However, it is difficult to assess whether such rearrangements were the causes or consequences of recombination suppression. As a matter of fact, large regions of recombination suppression without any inversions between mating types also have been identified in *Microbotryum* spp. (Branco *et al*., 2017; Carpentier *et al*., 2019), *P. anserina* (Grognet *et al*., 2014) and *N. tetrasperma* fungi (Y. Sun *et al*., 2017) (Fig. 6c), indicating that other mechanisms can suppress recombination.

A third, nonexclusive hypothesis relates to epigenetic modifications of chromatin structure associated with transposable elements (TEs) (Kent *et al*., 2017) (‘spread of transposable element hypothesis’; Fig. 8c; Table 1). TEs accumulate in regions of suppressed recombination (Bachtrog, 2003, 2013; Branco *et al*., 2018), where selection against their deleterious insertions is less effective than in freely recombining regions. Genome defenses against TE proliferation often involve the silencing of gene expression through constitutive heterochromatin assembly, sometimes driven by DNA methylation (Maloisel & Rossignol, 1998; Ben‐Aroya *et al*., 2004; Lewis *et al*., 2009; Yelina *et al*., 2012; Montanini *et al*., 2014; Li *et al*., 2016). The effects upon local genomic architecture often are not limited to the TE sequences but can extend, in terms of both DNA methylation and heterochromatin formation, up to several kilobases away. Thus, TE accumulation and/or their silencing may spread into adjacent regions, possibly driving a feedback of extending recombination suppression and the further favoring of TE accumulation in those regions (Willing *et al*., 2015; Kent *et al*., 2017; Choi & Lee, 2020). In addition, repeat‐induced point mutation (RIP), a mechanism specifically mutating repeated sequences in fungi, also has the consequence to decrease recombination rates, because it decreases similarity between homologous genomic regions containing repeats and because it induces the formation of constitutive heterochromatin (Aramayo & Selker, 2013). RIP has been found in the nonrecombining region around mating‐type genes (Hood *et al*., 2005; Grognet *et al*., 2014). RIP and TE methylation have further been shown to ‘leak’ further than just transposable elements (Van de Wouw et al., 2010e Wouw et al., 2010; Meunier *et al*., 2018). Here, the silencing mechanisms of transposable elements constitute proximate causes of recombination suppression whereas the evolutionary cause is the lack of effective selection against TE insertions and the selection for genomic defense against repeat multiplication.

Other putative proximate mechanisms of recombination suppression (Table 1) include chromatin compaction and subsequent depletion of recombination hotspots (Furman *et al*., 2020), that could be involved for example in cases of selection for recombination suppression for sheltering deleterious alleles. For instance, the presence of histone H3 lysine 9 methylation (H3K9me being the hallmark of constitutive heterochromatin) and RNA interference was associated to recombination suppression in the yeast fungal model *Schizosaccharomyces* (Ellermeier *et al*., 2010; Okita *et al*., 2019). Localized recombination modifiers also can act in *cis* or in *trans*. The recombination landscape was found to be highly contrasted between *A. bisporus* varieties, with the variety *bisporus* showing crossing‐overs only at chromosome ends and the variety *burnettii* showing a more homogeneous crossing‐over distribution (Sonnenberg *et al*., 2017), suggesting recombination suppression expansion. The intermediate recombination landscapes in hybrids and backcross (Foulongne‐Oriol *et al*., 2011) led to the hypothesis that the recombination suppression in central parts of chromosomes was a quantitative trait and thus under the control of multiple genetic loci (Sonnenberg *et al*., 2017). A quantitative trait loci (QTL) mapping analysis in fact revealed two QTLs located on chromosome l (i.e. the mating‐type chromosome) and three other QTLs located on chromosomes IV, VI and VII, respectively (Telgerd, 2017), suggesting the existence of several recombination modifiers, some of which at least acting in *trans*.

## VIII. Conclusion and future directions

Recent progress in sequencing technologies has revealed increasing number of cases of recombination suppression around fungal mating‐type genes whereas their evolutionary and proximate causes have been little explored. We hope that the recent findings highlighted here will foster further studies on the recombination suppression around mating‐type loci in fungi, to assess the generality of these patterns and contribute to our understanding of the evolutionary genomics of reproductive compatibility across a broader range of organisms. Mating‐type loci in pseudo‐homothallic and selfing fungi will be particularly interesting to investigate, but we definitely need to accumulate data on a more diverse range of fungal life cycles to be able to draw general patterns about the features associated with recombination suppression. Fungi represent excellent models to test general hypotheses about the causes of recombination suppression, thanks to their small and compact genomes, the decoupling of mating types from gamete size differences and their experimental tractability. Mating‐type loci in other organisms, in particular the fungal‐like oomycetes, also will be interesting subjects on which to study these hypotheses, as they have just begun to be discovered, and there seems to be gradual decrease in heterozygosity when going farther from the mating‐type locus* (Dussert *et al*., 2020).

Hypotheses could be tested by investigating, in a wide range of species, the patterns of accumulation of deleterious mutations, linkage disequilibrium, polymorphic rearrangements, transposable elements and methylation marks, especially at the margin of nonrecombining regions (Fig. 8). Distributions of crossovers, double‐strand breaks and chromatin modifications also need to be further investigated in fungal mating‐type chromosomes*. Furthermore, some of the transposable element and deleterious allele hypotheses predict a gradual extension of recombination suppression, whereas the chromosomal inversions or rearrangements predict large discrete ‘strata’, such that investigating the divergence patterns between mating types may allow disentanglement of various hypotheses. When more data are available on the extent of recombination suppression around mating‐type loci in fungi, contrasting features may further allow disentanglement of hypotheses by evolutionary comparative methods. The tractability of fungi as genetic models and the progress of technology to access to structural and epigenetic variation in genomes will likely enhance further discoveries in the near future.

## Author contributions

TG and FEH drafted the manuscript; TG, FEH, MD and FC designed the figures; and all authors contributed to the text.
